# DACPGTN: Drug ATC Code Prediction Method Based on Graph Transformer Network for Drug Discovery

**DOI:** 10.3389/fphar.2022.907676

**Published:** 2022-06-01

**Authors:** Chaokun Yan, Zhihao Suo, Jianlin Wang, Ge Zhang, Huimin Luo

**Affiliations:** ^1^ School of Computer and Information Engineering, Henan University, Kaifeng, China; ^2^ Henan Key Laboratory of Big Data Analysis and Processing, Henan University, Kaifeng, China

**Keywords:** drug ATC code, multi-label classification, interaction information, drug discovery, graph transformer network

## Abstract

The Anatomical Therapeutic Chemical (ATC) classification system is a drug classification scheme proposed by the World Health Organization, which is widely used for drug screening, repositioning, and similarity research. The ATC system assigns different ATC codes to drugs based on their anatomy, pharmacological, therapeutics and chemical properties. Predicting the ATC code of a given drug helps to understand the indication and potential toxicity of the drug, thus promoting its use in the therapeutic phase and accelerating its development. In this article, we propose an end-to-end model DACPGTN to predict the ATC code for the given drug. DACPGTN constructs composite features of drugs, diseases and targets by applying diverse biomedical information. Inspired by the application of Graph Transformer Network, we learn potential novel interactions among drugs diseases and targets from the known interactions to construct drug-target-disease heterogeneous networks containing comprehensive interaction information. Based on the constructed composite features and learned heterogeneous networks, we employ graph convolution network to generate the embedding of drug nodes, which are further used for the multi-label learning tasks in drug discovery. Experiments on the benchmark datasets demonstrate that the proposed DACPGTN model can achieve better prediction performance than the existing methods. The source codes of our method are available at https://github.com/Szhgege/DACPGTN.

## 1 Introduction

Drug research and development is time-consuming and costly. A new drug, from development to launch, takes decades of research and hundreds of millions of dollars. How to find new indications from existing approved drugs and reduce the cost of research discovery is a hot field in bioinformatics (Pushpakom et al., 2019; Jarada et al., 2020). The World Health Organization has established a complete drug classification system, Anatomical Therapeutic Chemical (ATC) (MacDonald and Potvin, 2004). Specifically, the standard ATC code in the ATC system can be used to represent drug class information, which facilitates the use of drugs during the treatment phase. When the ATC code of a drug compound is known, can be inferred its active ingredient, therapeutic, pharmacological, and chemical properties. Therefore, predicting the ATC code of a drug helps to use the drug correctly or identify novel potential indications, and speed up the drug development process, which is a common idea for drug repositioning research. (Hutchinson et al., 2004). The ATC code system divides drugs into five levels, based on the first-level of ATC codes, drugs are classified into 14 anatomical classes including Alimentary tract and metabolism, Blood and blood forming organs, Cardiovascular system, Dermatologicals, Genitourinary system and sex hormones, Systemic hormonal preparations, excluding sex hormones and insulins, Anti-infectives for systemic use, Antineoplastic and immunomodulating agents, Musculoskeletal system, Nervous system, Antiparasitic products, insecticides and repellents, Respiratory system, Sensory organs, Various. For a drug, it may belong to more than one class in first-level at the same time.

There are a large number of drugs without ATC codes in widely used drug information databases. ATC code prediction of new or existing drugs using traditional experimental methods is cumbersome and time-consuming. The development and application of machine learning provide the possibility to realize the rapid classification of drugs ATC code (Dunkel et al., 2008; Wu et al., 2013). In recent years, some multi-label classification methods have been proposed for drug ATC Code prediction. Chen et al. (2012), firstly proposed a method to classify drug ATC code by integrating drug chemistry-chemistry interaction information and chemistry-chemistry similarity information, and constructed benchmark dataset for the first-level code prediction of drug ATC code. Based on this benchmark dataset, some classification methods integrating multiple drug information to predict drug ATC codes are proposed. Cheng et al. (2017b) proposed a multi-label Gaussian kernel regression classifier named iATC-mISF. Based on medicinal chemical–chemical interaction, structure, and fingerprint similarity, assign the first-level ATC code to drugs. After that, Cheng et al. (2017a) improved the classifier’s performance by further integrating the predictor iATC-mDO based on the drug ontology information (Degtyarenko et al., 2007). Based on this, iATC-mISF has been upgraded to iATC-mHyb. Nanni and Brahnam (2017) developed a multi-label classifier EnsLIF based on gradient histogram algorithm, which constructs the one-dimensional feature vector of drug compounds into a two-dimensional matrix. Zhou et al. (2020a) constructed multiple drug interaction networks, extracted the drug features in the network through the network embedding algorithm Mashup (Cho et al., 2016), and transformed the original multi-label classification problem into multiple binary classification problems by using Random k-labelsets (RAKEL) algorithm (Tsoumakas and Vlahavas, 2007). In the classification stage, the classical machine learning algorithm support vector machine (SVM) (Cortes and Vapnik, 1995) is used to construct the classifier iATC-NRAKEL, which has achieved good results. Based on the iATC-NRAKEL classifier, Zhou et al. (2020b) proposed a multi-label classifier iATC-FRAKEL only used the fingerprints of drugs as feature. In addition, web services are provided. By integrating drug-drug interaction information, structural similarity, and fingerprint similarity, and using the NLSP method (Szymański et al., 2016) to explore the correlation between labels. Wang et al. (2019b) proposed a method ATC-NLSP, to predict the first-level ATC code of drugs, which uses a machine learning framework to provide better prediction results.

With the successful application of deep learning technology in many fields, Nanni et al. (2020) proposed a first-level ATC code multi-label classifier system (FUS3) by integrating multiple deep learning methods. The model used convolutional neural network (CNN) and Long-Short-Term Memory network (LSTM) (Hochreiter and Schmidhuber, 1997) to extract implicit features, then train two calssifiers to identify the ATC codes of drugs using extracted features. In the latest study, Zhao et al. (2021) proposed a new drug ATC code end-to-end prediction model CGATCPred, which utilized a multi-layer Convolutional Neural Network (CNN) to extract composite features from multiple types of drug features. The association graph structure of ATC code labels is established and combined with the word embedding information, the GCN (Kipf and Welling, 2016) network is applied to extract the label information. New features were obtained based on composite features and the generated label information. The generated features were spliced with the composite features extracted from the CNN layer, and then were input to the fully connected neural network layer to predict the ATC code of the drugs.

For the ATC code prediction problem, most of the existing classification methods generally consider the information of the drug itself or the relationships between the ATC code and drugs. These approaches ignore the potential importance of other relevant information in drug ATC code prediction, such as target protein and disease information associated with drugs. Several studies have demonstrated that similar drugs have similar in chemical properties, indications, etc (Chiang and Butte, 2009; Li and Lu, 2012). Based on this property, the general hypothesis is that when two drugs act on the same target protein or disease, or they have multiple interactions between two drugs and target protein or disease, they may have the same ATC code labels.

In this article, to improve the performance of drug ATC code identification, we proposed a novel drug ATC code prediction method based on the Graph Transformer Network (Yun et al., 2019). Traditional deep learning frameworks have some limitations (Zhang et al., 2018; Wang et al., 2019a). For example, it cannot effectively exploit the interaction information in heterogeneous networks or requires predefined fixed interactions between nodes. GTN model is a self-learning method for heterogeneous graphs. It uses graph transformer layer to learn potential interactions information between different nodes from multiple heterogeneous graphs (Shi et al., 2016) and apply learned information to node classification tasks. The crucial idea of GTN is heterogeneous network representation learning, which is suitable for exploring the interaction between different types of nodes is helpful for the performance improvement of classification tasks. For drug ATC code prediction, we integrate drugs and drug-related biomedical entities including targets and diseases. Then, we use the known interactions information to construct a set of heterogeneous networks, which contains information about different nodes. GTN model can be used to find potential interactions between different entities from these constructed heterogeneous networks, and these potential interactions can help to predict the ATC code of drugs. Therefore, a new first-level drug ATC code prediction model DACPGTN is proposed based on GTN.

DACPGTN predicts the first-level ATC code for a given drug by applying biomedial features and interactions of drugs, diseases and targets. In the study, drug-drug similarity information was obtained by integrating different types of compound interactions. Meanwhile, the similarity information of drug-related target proteins and diseases is calculated based on the known interactions between biomedical entities. The similarity information was used to construct a composite feature matrix. Next, we consider the introduction of drug-target protein, drug-disease and target protein-disease interactions information. Based on the known interactions information, a set of interaction heterogeneous networks between different biomedical entities are constructed. Then, the graph structure of the potential interactions information between drug-target protein-disease can be obtained by using the graph transformer layer. Finally, the composite feature matrix and the learned potential interactions information networks are fed into the prediction module for learning. According to the above steps, we can obtain the final prediction of the drug ATC code. Experiments on the benchmark datasets demonstrate that the DACPGTN model can achieve better prediction performance than the existing methods.

The main contributions of this article are as follows:

1) For the drug ATC code prediction task, the DACPGTN model considers the impact of integration drug-related biomedical entity information including target proteins and diseases on drug ATC code prediction performance.
2) By utilizing graph transformer network and multiple heterogeneous networks, DACPGTN learns potential valuable interactions information for identifying ATC code for drugs.
3) In this study, the GTN model is improved to address the problem of drug ATC code prediction. The previous research transformed the drug ATC code prediction problem into multiple independent binary classification problems (Kumari and Srivastava, 2017). By using cross-entropy loss function and softmax function, we improved the GTN model and solved the class-imbalance and complex parameter settings for model training. Moreover, prediction performance can be improved by using linear layers and adding Dropout layers between layers.

## 2 Materials

### 2.1 Dataset

#### 2.1.1 Drugs and Anatomical Therapeutic Chemical Codes

**FIGURE 1:**
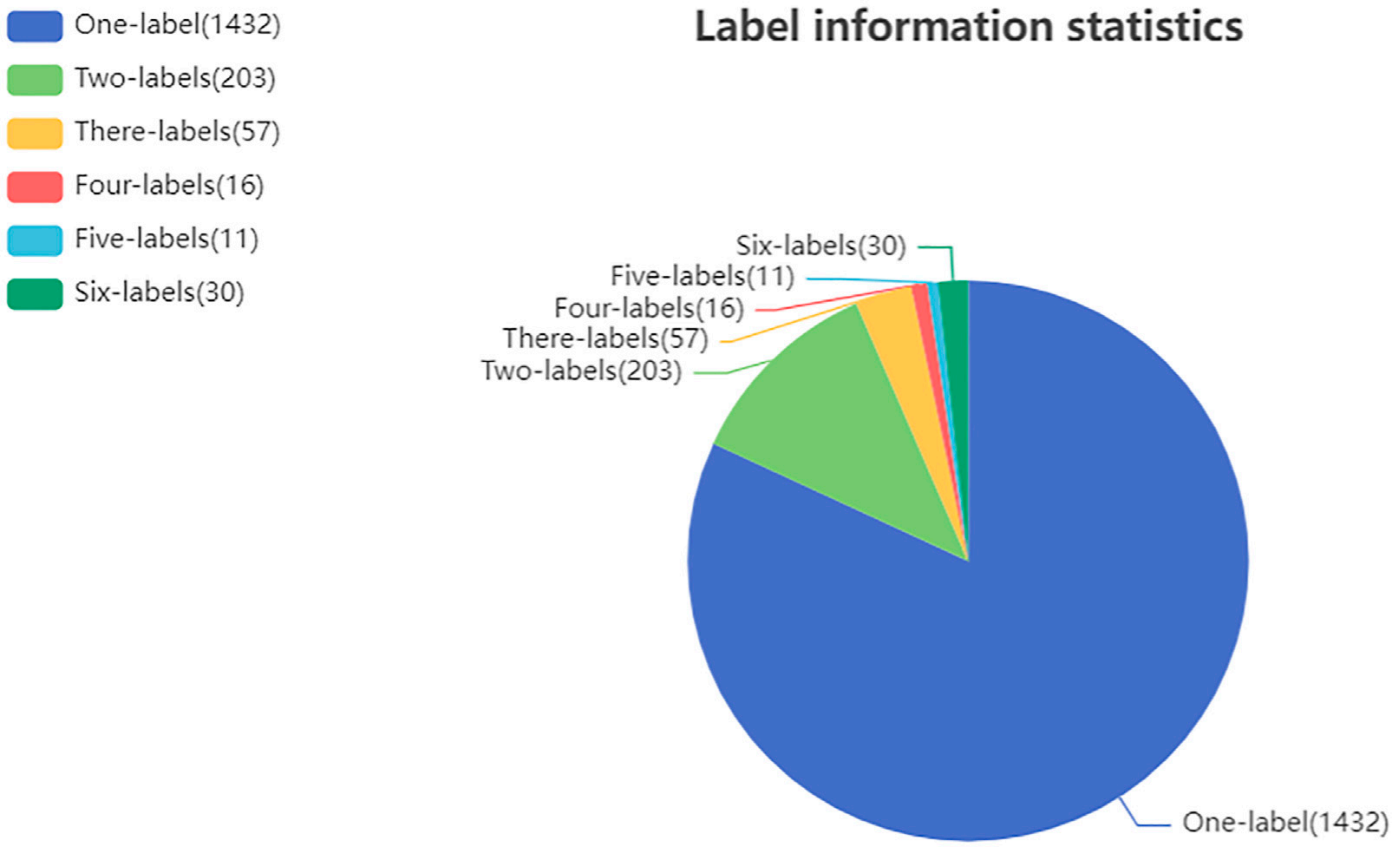
Benchmark dataset label information analysis.

For the ATC Code prediction problem, Chen et al. (2012) constructed benchmark dataset to facilitate comparison of models at the first-level of the ATC code. The benchmark dataset contains 3,883 drugs with one or more first-level classes of the ATC code. Moreover, we have collected drug related target proteins and diseases from the KEGG (Kanehisa and Goto, 2000) and Drugbank (Wishart et al., 2008), which are publicly available databases involving substantial data describing drugs, diseases, target proteins and interactions among them. Filtering the collected data revealed that 1,749 out of 3,883 drugs have target or disease information. Then, these 1,749 drugs were used as the benchmark dataset in our experiments. In this study, the prediction of a drug’s first-level ATC code is formulated as a multi-label problem (Tsoumakas and Katakis, 2007). For each given drug, it may have two or more labels to annotate its classification. The statistics for ATC code label information of all drugs in our dataset is shown in Figure 1.

Meanwhile, the dataset can be represented as a set of elements as: *S* = *S*_1_ ∪ *S*_2_ ∪ *S*_3_⋯ ∪ *S*_13_ ∪ *S*_14_, where *S*_*i*_ represents drugs in the *i*th class. Let *D*_*i*_ represents the *i*th drug, and $j\in \left\{1,\;2,\;\cdots \;,\;14\right\}$ represents the label of drug-class. The 1,749 drug compounds in the dataset can be classified into 14 ATC classes, as shown in Table 1. The ATC code labels for each given drug can be represented by a 14-bit binary vector defined as $Lable\left(D_{i}\right)=\left[L_{i1},\;L_{i2},\;L_{i3},\cdots \;,\;L_{\mathrm{i13}},\;L_{\mathrm{i14}}\right]\;\;\left(i=1,2,3,\ldots ,1749\right)$ . Where *L*_*ij*_ represents the relationship between drug *D*_*i*_ and first-level ATC code class *j*. The value of *L*_*ij*_ is defined as follows:

$$L_{ij}=\left\{\begin{array}{c} \begin{array}{cc} 1 & if\;\;Drug\;\;i\;\;belongs\;\;to\;\;class\;\;j \end{array}\;\\ \begin{array}{cc} 0 & else \end{array}\; \end{array}\right.$$

**TABLE 1:** The 1749 drug compounds in the benchmark dataset are broken down into 14 ATC classes.

Subset	Name	Number of Drugs
*S*_1_	Alimentary tract and metabolism	221
*S*_2_	Blood and blood forming organs	44
*S*_3_	Cardiovascular system	287
*S*_4_	Dermatologicals	182
*S*_5_	Genitourinary system and sex hormones	127
*S*_6_	Systemic hormonal preparations, excluding sex hormones and insulins	68
*S*_7_	Anti-infectives for systemic use	273
*S*_8_	Antineoplastic and immunomodulating agents	129
*S*_9_	Musculo-skeletal system	91
*S*_10_	Nervous system	382
*S*_11_	Antiparasitic products, insecticides and repellents	48
*S*_12_	Respiratory system	189
*S*_13_	Sensory organs	222
*S*_14_	Various	45
Number of total virtual drugs		2308^a^
Number of total structural different drugs		1749

a The number of virtual drugs is calculated as follows: when a drug belongs to two different classes at the same time, it is counted as two virtual drugs. If a drug belongs to three different classes at the same time, it is counted as three virtual drugs, and so on.

#### 2.1.2 Drug Targets and Indications

As mentioned above, the target proteins and diseases associated with 1749 drugs in the experiment were extracted from KEGG (Kanehisa and Goto, 2000) and Drugbank (Wishart et al., 2008), the two most widely used drug information databases. Specifically, the drug-related target proteins in the experiment were obtained from Drugbank, and we pre-processed the available information using the conversion tool provided on the Uniprot website to obtain 982 targets associated with the 1,749 drugs. Then, the drug-related diseases in the experiment were obtained from the KEGG database, and based on the known interactions information, a total of 355 related diseases were obtained. Table 2 summarizes the dataset in terms of numbers of drugs, target proteins, and diseases, as well as the interactions among them.

**TABLE 2:** Statistics of the Benchmark standard dataset used in this study.

Dataset	Drugs	Targets	Diseases
	1749	982	355
Interactions	Drug-Target	Drug-Disease	Target-Disease
	6,370	1,285	288

### 2.2 Construction of Similarity Matrix and Heterogeneous Networks

#### 2.2.1 Drug, Target, and Disease Similarity Matrix

In this study, seven types of drug-drug similarity information for 1749 drugs extracted from the previous literature (Zhao et al., 2021). *SM*_*Sim*_, *SM*_Exp_, *SM*_*Dat*_, *SM*_*Tex*_, *SM*_*Com*_ were obtained from the interaction information of “similarity”, “experimental”, “database”, “text mining” and “Combined score” between drug pairs. *SM*_*cp*_ and *SM*_*sub*_ were obtained using the compound similarity calculation tools SIMCOMP and SUBCOMP provided by the KEGG dataset. A single data source may be incomplete or limited, and it is extremely important to integrate various biomedical data from multiple sources in practice (Luo et al., 2021). Data integration helps to improve the accuracy of the data and the performance of drug repositioning, and we used averaging operations on the seven similarity matrices to obtain the final drug-drug similarity score matrix *M*_*RR*_.

For the 982 target proteins used in the experiments, combined score between proteins were obtained from the String library (Szklarczyk et al., 2019) to construct a protein-protein interaction score matrix. The combined score represents interaction strength between the two proteins. The larger the combined score, the stronger the interaction between the two proteins. After processing with the min-max normalization method, protein-protein similarity scores matrix *M*_*TT*_ is obtained.

Based on the hypothesis that similar drugs may treat similar diseases, we integrated disease similarity information for identifying the key features of drugs to assist the ATC code prediction in our study. Disease similarity is calculated by utilizing known interaction information between diseases and drugs (Luo et al., 2016). Specifically, for the 355 diseases in our experiments, we construct a drug-disease interactions matrix by using all drugs in the Chen et al. (2012) benchmark dataset. As for this drug-disease interactions matrix, if there exists an interaction between drug *R*_*i*_ and disease *D*_*j*_, the edge weight of *R*_*i*_ and *D*_*j*_ is initially assigned as 1 and otherwise 0. Finally, the Pearson correlation coefficient (Benesty et al., 2009) of the matrix is calculated to obtain the disease-disease similarity matrix *M*_*DD*_.

#### 2.2.2 Drug-Target-Disease Heterogeneous Networks

We collected the known interactions information of the three biomedical entity nodes of drugs, target proteins, and diseases in the KEGG and Drugbank databases. The known interactions information is used to construct the corresponding heterogeneous network.

More specifically, we let $R=\left\{R_{1},R_{2},\cdots \;,R_{m}\right\}$ denotes *m* drugs, $T=\left\{T_{1},T_{2},\cdots \;,T_{q}\right\}$ denotes the *q* targets and $D=\left\{D_{1},D_{2},\cdots \;,D_{n}\right\}$ denotes the *n* disease. The drug-target network contains *m* drugs and *q* targets, if there exists an interaction between drug *R*_*i*_ and target *T*_*j*_, the edge weight of *R*_*i*_ and *T*_*j*_ is initially assigned as 1 and otherwise 0. Likewise, the drug-disease network includes *m* drugs and *n* diseases, if there exists an interaction between drug *R*_*i*_ and disease *D*_*j*_, the edge weight of *R*_*i*_ and *D*_*j*_ is initially assigned as 1 and otherwise 0. Meanwhile, the target-disease network consists of *q* targets and *n* diseases, if there exists an interaction between target *T*_*i*_ and disease *D*_*j*_, the edge weight of *T*_*i*_ and *D*_*j*_ is initially assigned as 1 and otherwise 0. *H*_*RT*_, *H*_*DD*_ and *H*_*TD*_ are defined as the interaction matrices of drug-target network, drug-disease network and target-disease network, respectively.

## 3 Drug Anatomical Therapeutic Chemical Code Prediction Model Based on GTN

**FIGURE 2:**
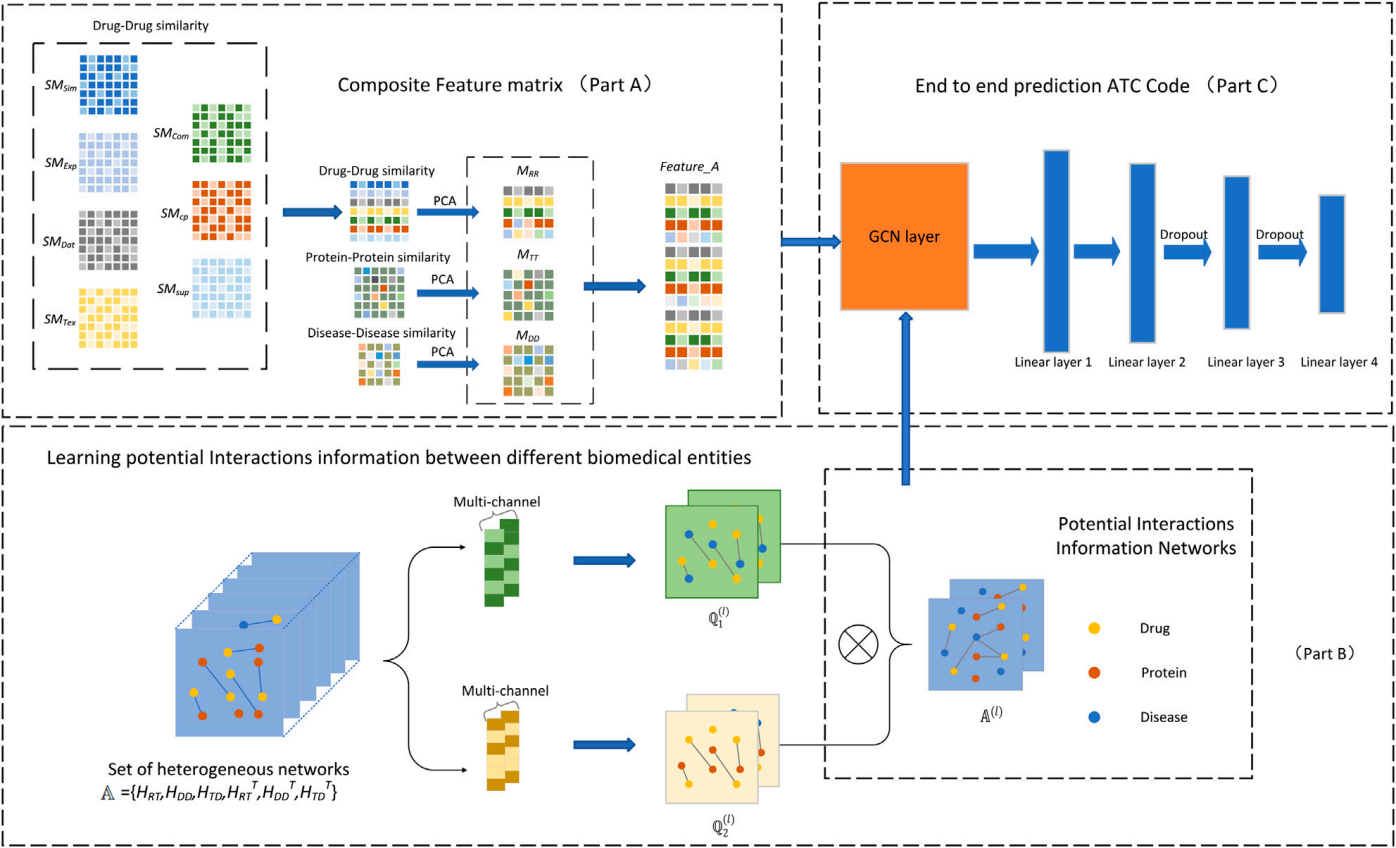
Overall framework of DACPGTN. The feature information of different biomedical entities is integrated to construct a composite feature matrix as the node feature input of the prediction module (Part A). The graph transformer layer is used to obtain the potential interactions information between different biomedical entities from heterogeneous networks set (Part B). The prediction stage uses the composite feature matrix and the learned Potential Interactions Information Networks to obtain prediction results (Part C).

In this study, we have proposed a DACPGTN model for multi-label prediction of drug ATC code based on the GTN model. We first integrate the drugs and their associated target proteins and diseases, and construct a composite feature matrix by using the similarity information of the three biomedical entities as features. Meanwhile, a set of heterogeneous networks are constructed based on the known interactions information between different biomedical entities. Based on the Graph Transformer Network (Yun et al., 2019), the potential interactions information between drug-target-disease is obtained from these heterogeneous networks, which has an impact on the prediction of drug ATC code. Then, the constructed composite feature matrix and the learned potential interactions information between biomedical entities are fed into the end-to-end prediction module to obtain the ATC code prediction results for a given drug. The overall framework of the DACPGTN model is shown in Figure 2.

### 3.1 Construction of Composite Feature Matrix

The similarity information of the three biomedical entities including drugs, targets and diseases is used to construct similarity matrix representing their features. Principal component analysis (PCA) (Abdi and Williams, 2010), commonly used technique for dimension reduction, is used to project drugs, targets and diseases into a low-dimensional space. Then, these low-dimensional matrices are unified to obtain the corresponding feature matrix. Using PCA can remove the noise data to a certain extent, maximize the retention features at the same time, provide valuable information for drug ATC code prediction. It is verified experimentally that the model has the best training effect when the dimension is 300. After unifying the feature dimensions, the feature matrices of the three biomedical entities are spliced to obtain the final node composite feature matrix $Feature\text{\_}A=\left[M_{RR};M_{TT};M_{DD}\right]$ (Part A of Figure 2).

### 3.2 Learning Potential Interactions Between Entities Based on Graph Transformer Layer

In this study, the graph transformer model is applied to learn valuable interactions information between drugs, targets and diseases from the heterogeneous networks constructed above. The constructed drug-target heterogeneous network, drug-disease heterogeneous network, and target-disease heterogeneous network are sequentially transposed and the dimensions are unified. Then, the set of heterogeneous networks $A=\left\{H_{RT},H_{DD},H_{TD},{H_{RT}}^{T},{H_{DD}}^{T},{H_{TD}}^{T}\right\}$ is obtained. The graph transformer layer is used for set A to obtain networks of potential interactions information between three biomedical entities: drugs, target proteins and diseases. The transfer of interaction information between nodes is achieved by multiplication operations between different associated heterogeneous networks (Wang et al., 2019a) (Part B of Figure 2).

Specifically, the graph transformer layer is used to perform a soft selection of different edge types and composite relations (Chen et al., 2018) to find new graph structures from multiple candidate heterogeneous networks. The graph transformer layer is implemented as formula (1):

$$Q=F\left(A;W_{\phi }\right)=\phi \left(A;softmax\left(W_{\phi }\right)\right)$$ (1)

Where *ϕ* is the convolution layer and *W*_*ϕ*_ ∈ **R** ^1×1×*K*^ is the parameter of the convolution layer *ϕ*.

The graph transformer layer selects different types of interaction matrices from the set A . Then, a new graph structure is learned by matrix multiplication of the selected interaction matrices *Q*_1_ and *Q*_2_. The soft selection of the interaction matrix refers to obtaining non-negative weights from $softmax\left(W_{\varphi }\right)$ , and perform 1 × 1 convolution weighted summation over the candidate matrices in the heterogeneous network set A . In the implementation process, the constructed interaction matrix is operated on graph transformer layer by Eq. 2–4, each *Q*_1_ can be expressed as $Q_{i}=\underset{t\in T^{e}}{\sum }\alpha_{t}^{\left(l\right)}A_{t}$ , $T^{e}$ represents the set of networks, *l* represents the *l*-th graph transformer layer, and $\alpha_{t}^{\left(l\right)}$ represents the weight of the current network matrix in the *l*th layer. The connection between different nodes is obtained by multiplication of different types of interaction matrices. For A , the graph transformer layer is used to learn potential interactions information between the three biomedical entities to obtain a new graph information matrix.

When the weight-based graph structure is obtained, the multiplication operation between the new graph structures is performed. To improve numerical stability, the interaction matrix obtained for each layer is normalized by its degree matrix *D* ^−1^.

$$Q_{1}=F\left(A;W_{\phi }\right)=\phi \left(A;softmax\left(W_{\varphi }^{1}\right)\right)$$ (2)

$$Q_{2}=F\left(A;W_{\phi }\right)=\phi \left(A;softmax\left(W_{\varphi }^{2}\right)\right)$$ (3)

$$A^{\left(l\right)}=D^{-1}Q_{1}Q_{2}$$ (4)

The graph transformer layer can also learn a variety of connection relationships between different node types. To learn multiple potential interaction information networks between biomedical entities simultaneously, we use *C* channels in parallel to accomplish this operation and add the identity matrix *I* to A for learning variable-length interaction information. By setting the output channels of the 1 × 1 convolution in the graph transformer layer to multi-channel *C*, the adjacency matrices *Q*_1_, *Q*_2_ become adjacency tensor $Q_{1}^{\left(l\right)},Q_{2}^{\left(l\right)}\in R^{N\times N\times C}$ . After stacking *l* graph transformer layers, the tensor $A^{\left(l\right)}\in R^{N\times N\times C}$ is obtained.

In order to discover potential interactions between different nodes to inform the label prediction of drug nodes, the graph transformation layer is applied to the heterogeneous network sets A to learn the node interactions information in each associated heterogeneous network. For example, according to the relationship of drug-target protein, target protein-disease, etc., we can learn the interactions between the drug and potential disease, such as $\left(Drug\overset{D\text{\_}T}{\to }Target\overset{T\text{\_}D}{\to }Disease\right)$ , etc.

### 3.3 Realization of End-To-End Prediction of DACPGTN Model

For the prediction module of the DACPGTN model, we use GCN as the feature extractor of the end-to-end module, and then take the node composite feature matrix and the learned potential interactions information as the input of the end-to-end prediction module. Embeddings of drug nodes are extracted through GCN, multiple linear layers and Dropout (Srivastava et al., 2014) layers are combined to predict the final drug ATC code. A novel loss function is introduced to complete the training of the model in this experiment. The detailed implementation process of the end-to-end prediction module is shown in Part C of Figure 2.

#### 3.3.1 Graph Convolutional Neural Network Learning on Composite Feature Matrix and New Graph Structure

Graph Convolutional Neural Network (GCN) (Kipf and Welling, 2016) is a semi-supervised learning algorithm, which is used for the convolutional operation of the associated information graph structure and the composite feature matrix. For the GCN network, layer-to-layer propagation is performed according to formula (5):

$$H^{\left(l+1\right)}=\sigma \left({\tilde{D}}^{-\frac{1}{2}}\tilde{A}{\tilde{D}}^{-\frac{1}{2}}H^{\left(l\right)}W^{\left(l\right)}\right)$$ (5)

Where $\tilde{A}$ is a new graph matrix generated by graph transformer layer, $\tilde{D}$ is the degree matrix of $\tilde{A}$ , *H* is the input feature of the current GCN network layer, that is, the constructed node feature matrix, *W* ^(*l*)^ ∈ **R** ^*d*×*d*^ is a trainable weight matrix, *H* ^(*l*+1)^ is the output of the feature matrix of the GCN network layer, and *σ* represents the activation function Relu. When the output channel of the graph transformer layer 1 × 1 convolution is set to multi-channel *C*, the GCN layer is applied to each channel of the tensor, and the multi-channel operation is performed through formula (6).

$$Z={∥}_{i=1}^{C}\sigma \left({\tilde{D}}_{i}^{-1}{\tilde{A}}_{i}^{\left(l\right)}XW\right)$$ (6)

Where ∥ represents the connection operator, *C* represents the number of output channels, ${\tilde{A}}_{i}^{\left(l\right)}=A_{i}^{\left(l\right)}+I$ represents the *i*th adjacency matrix of the tensor $A^{\left(l\right)}$ add the identity matrix *I*, ${\tilde{D}}_{i}$ represents the degree matrix of $A_{i}^{\left(l\right)}$ , *W* ∈ **R** ^*d*×*d*^ represents the trainable cross-channel shared weight matrix, *X* ∈ **R** ^*N*×*d*^ represents the feature matrix *Feature*_*A*, *N* and *d* represent the number of biomedical entity nodes and the node features dimension in *Feature*_*A*, respectively.

The GCN network obtain dimension-specific drug node embeddings after a convolution operation on the node feature matrix *Feature*_*A* and the adjacency tensors $A^{\left(l\right)}$ . For the case of networks with few nodes, it has been shown in the literature that if a GCN network is stacked with multiple layers, the output features may be over-smoothed and vertices from different clusters may become indistinguishable (Li et al., 2018; Li et al., 2019). In this study, limited by few nodes, the GCN network used in the feature extraction module has only one layer. The drug nodes embedding extracted by the GCN network is used as the input information for the next part of the linear layers.

#### 3.3.2 Transformation of Multi-Label Problem

As a multi-label classification problem, drug ATC code prediction differs from the traditional single-label multi-classification task. It requires that the prediction output of the model is not a fixed value. For a given drug, it may have one or more labels representing its classification information at the same time, which further increases the requirements of the classifier. For this problem, the common idea of previous research is to transform the multi-label classification problem into multiple independent binary classification problems. Each binary classification problem corresponds to a label in the label vector and determines the drug’s ATC code. For multiple independent binary classification problems, the sigmoid activation function with binary classification cross-entropy loss (BCEloss) is used to average the loss of all binary classifications, which is applied to model training to obtain the final prediction result. When the real class of the sample is far less than the number of all classes of the problem, there will be a class-imbalance problem, and some balance strategies are generally used to solve this problem. For example, setting a threshold for each binary classification problem or manually adjusting the weights of positive and negative samples, etc. To simplify the complex series of operations after transforming a multi-label problem into multiple independent binary classification problems, we refer to Su’s use of Circle loss (Su, 2020; Sun et al., 2020). The softmax activation function is combined with the Cross-Entropy Loss function for multi-label classification problems. The implementation is as follows:

In a single-label classification problem, assuming that the scores of each class are $\left\{S_{1},S_{2},\ldots ,S_{n-1},S_{n}\right\}$ , and the target class is *t* ∈ {1, 2, … , *n*}, its cross-entropy loss function is defined as formula (7):

$$-\log\frac{e^{S_{t}}}{\sum_{i=1}^{n}e^{S_{i}}}=-\log\frac{1}{\sum_{i=1}^{n}e^{S_{i}-S_{t}}}=\log\overset{n}{\underset{i=1}{\sum }}e^{S_{i}-S_{t}}=\log\left(1+\overset{n}{\underset{i=1,i\neq t}{\sum }}e^{S_{i}-S_{t}}\right)$$ (7)

It can be derived as an approximation of the max function as shown in formula (8):

$$\log\left(1+\overset{n}{\underset{i=1,i\neq t}{\sum }}e^{S_{i}-S_{t}}\right)\approx \max\left\{\begin{array}{c} 0\\ s_{1}-s_{t}\\ \vdots \\ s_{t-1}-s_{t}\\ s_{t+1}-s_{t}\\ \vdots \\ s_{n}-s_{t} \end{array}\right\}$$ (8)

In this loss, all non-target class scores $\left\{S_{1},\cdots \;,S_{t-1},S_{t+1},\cdots \;,S_{n}\right\}$ are compared with target class scores *S*_*t*_ and their maximum difference should be less than zero, thus ensuring that target class score is greater than each non-target class score. In the multi-label classification problem, we also want each target class score to be no less than the score of each non-target class, and the generalization of Loss is obtained according to the same principle (Sun et al., 2020), as formula (9):

$$\log\left(1+\underset{i\in \Omega_{\mathrm{neg}},j\in \Omega_{\mathrm{pos}}}{\sum }e^{s_{i}-s_{j}}\right)=\log\left(1+\underset{i\in \Omega_{\mathrm{neg}}}{\sum }e^{s_{i}}\underset{j\in \Omega_{\mathrm{pos}}}{\sum }e^{-s_{j}}\right)$$ (9)

Where Ω_*pos*_ and Ω_*neg*_ are the set of target and non-target classes for a given sample in the multi-label problem, respectively.

When the samples have a fixed number of labels *k* in a multi-label classification problem, the above formula can be used directly to output the *k* classes with the top score in the prediction stage. In the actual multi-label prediction problem, the number of labels *k* owned by the sample is a constant with non-fixed value, and a threshold is needed to determine all classes of the sample. To this end, an additional class of *S*_0_ is introduced, and it is desired that all scores of the target class are greater than *S*_0_ and all scores of the non-target class are less than *S*_0_, which is obtained as formula (10):

$$\log\left(1+\underset{i\in \Omega_{\mathrm{neg}},j\in \Omega_{\mathrm{pos}}}{\sum }e^{s_{i}-s_{j}}+\underset{i\in \Omega_{\mathrm{neg}}}{\sum }e^{s_{i}-s_{0}}+\underset{j\in \Omega_{\mathrm{pos}}}{\sum }e^{s_{0}-s_{j}}\right)=\log\left(e^{S_{0}}+\underset{i\in \Omega_{\mathrm{neg}}}{\sum }e^{s_{i}}\right)+\log\left(e^{-s_{0}}+\underset{j\in \Omega_{\mathrm{pos}}}{\sum }e^{-s_{j}}\right)$$ (10)

Setting the threshold *S*_0_ to the default value of 0, we can get the simplified formula (10) of formula (11):

$$\log\left(1+\underset{i\in \Omega_{\mathrm{neg}}}{\sum }e^{s_{i}}\right)+\log\left(1+\underset{j\in \Omega_{\mathrm{pos}}}{\sum }e^{-s_{j}}\right)$$ (11)

The final Loss is obtained as a generalization of the softmax activation function with the cross-entropy loss function on the multi-label classification problem, as formula (12):

$$loss\left(y_{\mathrm{true}},y_{\mathrm{pred}}\right)=\;logsumexp\left(y_{\mathrm{pred-neg}},0\right)+logsumexp\left(y_{\mathrm{pred-pos}},0\right)$$ (12)

In this experiment, formula(12) is used to calculate the loss. Once the loss is obtained, backpropagation is performed to train the model. In the prediction stage of the model, classes with target scores greater than 0 are output. Compared with the methods in previous ATC code prediction studies, the multi-label problem is no longer transformed into multiple binary classifications, but into the comparison of target class scores and non-target class scores. In the optimization process, the logsumexp function (Blanchard et al., 2019) automatically takes part with the largest loss for learning. The logsumexp function will reduce the weight of the items that have been optimized well, and highlight the items with larger errors, and the class-imbalance problem is solved to some extent.

#### 3.3.3 Predicting Drug Anatomical Therapeutic Chemical Code

After extracting the feature embedding of nodes through the GCN(Kipf and Welling, 2016) network, we further process the embedding of drug nodes using linear layers and Dropout (Srivastava et al., 2014) layers to obtain better drug ATC code prediction performance. Specifically, the drug nodes embedding extracted by the GCN module is used as the input of the first linear layer. The output dimension of the last linear layer is the same as the dimension of the drug ATC label vector, which is used as the prediction result of the drug ATC code, and the model is optimized using the loss function introduced above. To solve the problem of multi-layer network stacking, a Relu activation function (Agarap, 2018) is used after the first linear layer, and Dropout layers are added between subsequent linear layers. The Dropout layer removes the neuron nodes from the network with a certain probability. In random gradient descent, the randomly removed neurons can make each iteration train a different network and increase the diversification of the network, thus improving the generalization ability of the model.

## 4 Experiments and Results

In this section, our experiments are performed on the benchmark dataset. First, the evaluation metrics used in this study are introduced. Then, the performance of DACPGTN is evaluated in comparison with several state-of-the-art drug ATC code prediction methods. Next, the effects of parameters and multiple sources of information on the DACPGTN model are analyzed through experiments.

### 4.1 Evaluation Metrics

For multi-label classification problems, since the samples have one or more labels at the same time, traditional single-label evaluation metrics are not applicable here. Compared with the traditional single-label evaluation metrics, the evaluation metrics for multi-label problems are more complex and complete. Five evaluation metrics for evaluating the performance of multi-label classifiers are defined in the literature published by Chou (Chou, 2013), and previous studies of the drug ATC label classification problem have used this evaluation criterion for comparison. To ensure the fairness of the experiments, we also use this evaluation criterion in our experiments. The definitions of the evaluation metrics are given in Equation (13–17):

$$Aiming=\frac{1}{N}\overset{N}{\underset{i=1}{\sum }}\left(\frac{Y_{i}\cap Y_{i}^{*}}{\left|Y_{i}^{*}\right|}\right)$$ (13)

$$Coverage=\frac{1}{N}\overset{N}{\underset{i=1}{\sum }}\left(\frac{Y_{i}\cap Y_{i}^{*}}{\left|Y_{i}\right|}\right)$$ (14)

$$Accuracy=\frac{1}{N}\overset{N}{\underset{i=1}{\sum }}\left(\frac{\left|Y_{i}\cap Y_{i}^{*}\right|}{\left|{Y_{i}\cup Y}_{i}^{*}\right|}\right)$$ (15)

$$Absolute-True=\frac{1}{N}\overset{N}{\underset{i=1}{\sum }}K\left(Y_{i},Y_{i}^{*}\right)$$ (16)

$$Absolute-False=\frac{1}{N}\overset{N}{\underset{i=1}{\sum }}\left(\frac{\left|{Y_{i}\cup Y}_{i}^{*}\right|-\left|Y_{\;i}\cap Y_{i}^{*}\right|}{M}\right)$$ (17)

where *N* is the total number of samples, *M* is the number of labels, the operator $\left|\cdot \right|$ is used to calculate the number of elements in the set, ∪/∩ represents the merge/intersection operation of the set, *Y*_*i*_ represents the true label vector of the current sample *i*, $Y_{i}^{*}$ represents the predicted label vector of the current sample *i* after the model, and *K* represents the function to determine whether the two vectors are identical, through formula (18):

KYi,Yi∗=10ifYi∗exactlythesameasYielse (18)

For our experiments, we used the 10-fold cross-validation (Refaeilzadeh et al., 2009) to evaluate the model’s performance. K-fold cross-validation is a rigorous evaluation method. In each fold, the dataset is divided into (training set: validation set): test set = (9:1):1. The performance of the model is evaluated by taking the average of 10 times repeated 10-fold cross-validations to ensure that the error in the experimental results is as small as possible.

### 4.2 DACPGTN Model Settings

This section lists the parameter settings of the experiment in Table 3. The learning rate is adapted by Adam optimizer (Zhang, 2018). This algorithm has an excellent performance in deep learning and has significant advantages compared with other types of random optimization algorithms. The model selection is based on the performance of the validation sets. We set the model training iterations for 250 epochs. Before each training, the performance of the current model on the validation set is compared with the performance of the previous epoch. Finally, select the model that achieves the best performance on the validation set to save. Setting of model parameters, based on the GTN model. The GCN network output dimensions that affect prediction performance are discussed in detail in Section 4.4. The overall DACPGTN model was implemented using the Python-based Pytorch 1.5.1 framework. These experiments were implemented on Windows 10 using python 3.6 and executed on a PC with a 2.90 GHz Intel Core i7-10700 processor and 32.0 GB RAM.

**TABLE 3:** DACPGTN model parameter settings.

Parameter	Detailed Settings
Number of Graph Transformer Layer	1
Number of channels	2
Training epochs	250
Learning rate	0.005
Weight decay	0.001
Number of GCN	1
Feature Input dim	300
GCN Output dim	150
FC1	150
FC2	128
FC3	64
FC4	14
Dropout	0.2

### 4.3 Comparison With Other Anatomical Therapeutic Chemical Code Multi-Label Classifiers

In this section, the DACPGTN model was compared with some of the state-of-the-art methods in drug ATC code prediction. We compared three state-of-the-art methods, 1) CGATCPred (Zhao et al., 2021), it uses a multi-layer convolutional neural network (CNN) to extract composite features from multiple types of drug-drug similarities, and uses a GCN network to learn the information between ATC Code labels. All the information learned is integrated and a neural network is used to make the final prediction. 2) iATC-NRAKEL (Zhou et al., 2020a), have constructed multiple drug-drug interaction networks, extracted the drug features by the network embedding algorithm Mashup. In the classification stage, the classic machine learning algorithm support vector machine was used. 3) iATC-mISF(Cheng et al., 2017b), a multi-label Gaussian kernel regression classifier. The first-level ATC Code for a given drug is predicted based on drug chemistry-chemistry interactions, drug structure similarity and drug fingerprint similarity. At the same time, in order to verify that deep learning method can provide better prediction performance than traditional multi-label classifiers, we also compare two basic multi-label classification methods ML-KNN(Szymanski and Kajdanowicz, 2017) and ML-RandomForest (Szymanski and Kajdanowicz, 2017). The parameter settings of all comparison models are the same as the optimal parameters in the original article, and the traditional classifier parameters are set as default. The comparative experiments are carried out on the dataset we constructed, and the results are shown in Table 4.

**TABLE 4:** Comparison with other ATC Code multi-label classifiers (10 × 10-fold CV).

Classfier	Aiming	Coverage	Accuracy	Absolute True	Absolute False
DACPGTN	0.8543	0.8517	0.8320	0.7902	0.0241
CGATCPred	0.7864	0.8022	0.7711	0.7290	0.0338
iATC-NRAKEL	0.7744	0.8020	0.7550	0.6947	0.0376
iATC-mISF	0.7094	0.7127	0.7036	0.6306	0.0244
ML-KNN	0.7293	0.7071	0.6861	0.6300	0.0433
ML-RandomForest	0.6723	0.6533	0.6471	0.6187	0.0368

**FIGURE 3:**
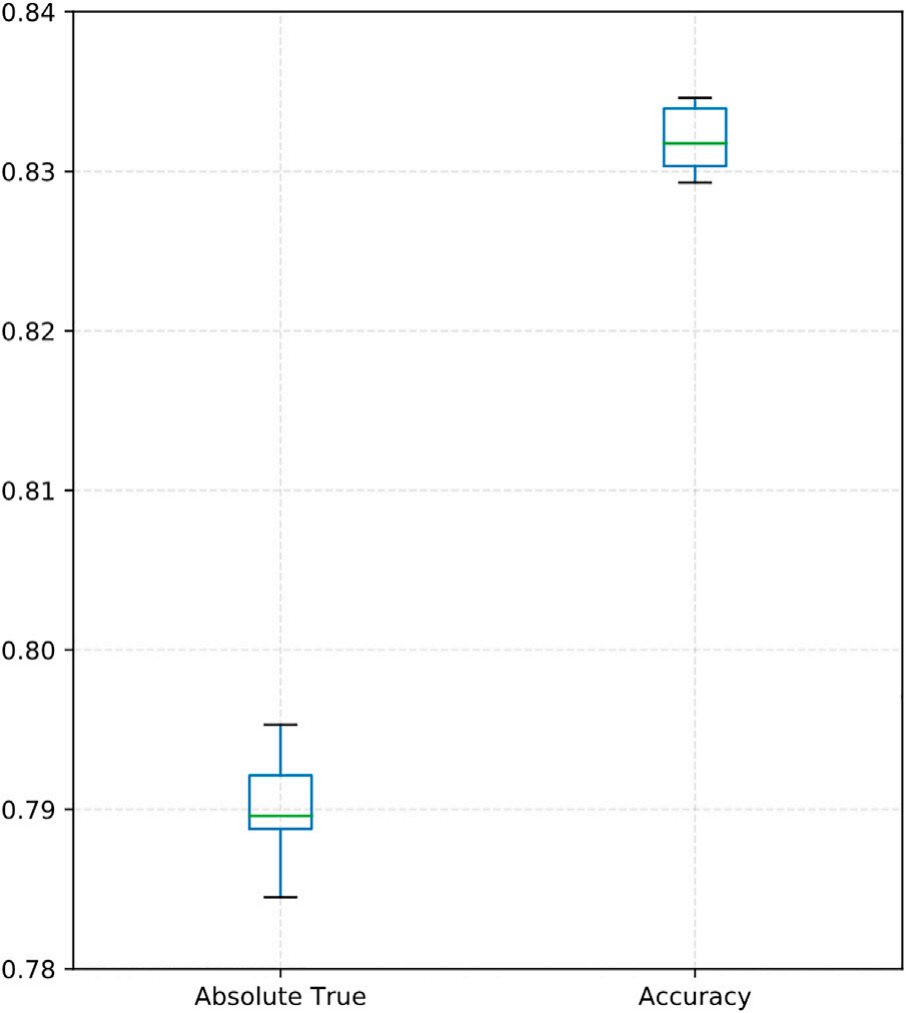
Boxplot showing the absolute trues and accuracies of DACPGTN with 10-fold cross-validation for 10 times.

As shown in Table 4, our proposed DACPGTN model has the best performance on the Benchmark dataset. Compared with the optimal model CGATCPred in drug ATC Code prediction problem, the improvement is 6.8% in Aiming,5% in Coverage,6% in Accuracy, and 6.1% in Absolute true. Accuracy and Absolute true are the most important among the five evaluation metrics (Qiu et al., 2016), and our model achieves a certain degree of improvement in these two metrics. To clearly show the performance of DACPGTN with 10 times repeated10-fold cross-validation, we illustrated a boxplot of accuracy and absolute true in Figure 3. The two measurements did not vary considerably, representing the stability of DACPGTN under different divisions of drugs. These results suggest that the DACPGTN model, which can learn potential interactions information between different biomedical entities from multiple heterogeneous graphs by using graph transformer layer. DACPGTN integrated potential interactions information and composite features between these nodes, which can achieve better performance in drug ATC code prediction.

### 4.4 The Effect of GCN Network Output Dimension

**FIGURE 4:**
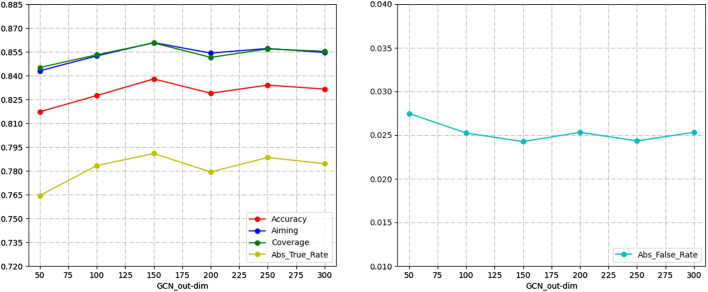
GCN network Output dimension selection.

In this experiment, the GCN network as a feature extractor provides classification information for the end-to-end prediction stage by learning the composite feature matrix and the potential interactions information matrix obtained from the graph transformer layer. In order to verify the effect of the GCN network node feature output size on the experimental results, the following experiments were conducted. The results are shown in Figure 4.

For the results of the experiments, we compare the performance of the GCN network in different output dimensions on five evaluation metrics. As shown in Figure 4, the model achieves the best prediction performance when the output dimension of the GCN network is 150. Therefore, the GCN network output dimension was set to 150, and all experiments were performed on this parameter.

### 4.5 The Effect of Multi-Source Interaction Information

To obtain drug-target protein interaction information and drug-disease interaction information on the impact of the drug ATC prediction problem. We used the drug-target protein interaction information and drug-disease interaction information as the input of the heterogeneous network, respectively, and reconstructed the feature matrix. The parameters of the experiments are the same as those in Section 4.2, and the results are shown in Table 5.

**TABLE 5:** Experimental results of single-source interaction information.

Classfier	Aiming	Coverage	Accuracy	Absolute True	Absolute False
DACPGTN-Disease	0.8442	0.8437	0.8231	0.7782	0.02516
DACPGTN-Target	0.8327	0.8307	0.8051	0.7536	0.02875

As shown in Table 5, the performance of the model degrades when only drug-target protein interaction information or only drug-disease interaction information is used as candidate adjacency matrix for heterogeneous networks. Meanwhile, only drug-target protein interaction information was used better than only drug-disease interaction information, and the experiment results were consistent with our expectation. Compared with single interaction information, the DACPGTN model obtained better prediction performance by considering multiple sources of interactions information. It is fully demonstrated that the DACPGTN model can extract useful information from multi-source interaction information for prediction. That is, new graph structures obtained by learning different heterogeneous graphs can contribute to the drug ATC code prediction problem.

### 4.6 Predicting Anatomical Therapeutic Chemical Code for New Drugs

To evaluate the capability of the DACPGTN model in predicting ATC Code for new drugs, we have conducted the following series of experiments. For a given new drug, it may not be possible to obtain information on its known targets or disease interactions. We consider three potential cases: 1) Drugs have interactions with targets. 2) Drugs have only interactions with diseases. 3) Drugs have no interactions with targets and diseases. For each potential case, we sequentially masked the interactions information for all drugs in the test set. The known interaction information of drugs in the heterogeneous network is removed, and the heterogeneous network set is reconstructed. Specifically, we set all elements of the row in the drug correspondence heterogeneous network to 0. When the known interactions information is removed, the given drug thus becomes a new drug with only drug-target interaction information or drug-disease interaction information or without any known interaction information. We performed the ten times repeated 10-fold cross-validation experiments for each case and took the average value to ensure that the error was sufficiently small. The experimental results are shown in Table 6. The experimental results show that the performance of the DACPGTN model decreases when the new drugs have different degrees of missing interaction information, but the performance of the model remains at a high level. This good performance may be related to the principle of the GCN network. When the test node learns fewer potential interactions by graph transformer layer or only self-interaction information, the GCN network can still transform the node features on the whole graph space. New drugs prediction experiments have demonstrated that the DACPGTN model has practical application. When a new drug is given, its target or disease interaction information is missing, or the interaction information between the new drug and these two types of biomedical nodes is unknown. We can still integrate existing heterogeneous networks to make well-performing ATC code predictions for new drugs using only drug-drug similarity information or partially known interactions information.

**TABLE 6:** New drugs prediction experiment results.

Interactions	Aiming	Coverage	Accuracy	Absolute True	Absolute False
None-Disease	0.8458	0.8443	0.8233	0.7802	0.0250
None-Target	0.8439	0.8423	0.8206	0.7764	0.0252
None-Target-Disease	0.8406	0.8376	0.8175	0.7747	0.0258

### 4.7 Case Studies

To further validate the reliability capability of DACPGTN, we selected some representative drugs for detailed case studies. Due to the early construction of the benchmark dataset and the limited information on drugs ATC code included, the DACPGTN model will give false positives of ATC code for some drugs in the prediction phase. As drug discovery research progresses, the pharmacological properties and ATC code of some drugs in the experimental dataset will be newly validated and supplemented. We have analyzed and validated some representative drugs predicted by our model with false positive ATC code through authoritative public databases, such as DrugBank (Wishart et al., 2008), CTD (Davis et al., 2021) and KEGG (Kanehisa and Goto, 2000). The predicted results and the supporting evidences are summarized in Table 7. For example, Brinzolamide (D00652) is a highly specific, non-competitive, reversible carbonic anhydrase inhibitor. It is indicated in the treatment of elevated intraocular pressure in patients with ocular hypertension or open-angle glaucoma. This drug was originally classified under the Sensory Organs, and new studies suggest it has been added to the cardiovascular class of the KEGG database. Carisoprodol (D00768) is a centrally acting skeletal muscle relaxant that does not act directly on skeletal muscle but acts directly on the central nervous system (CNS). Overdose of carisoprodol can depress the CNS and in severe cases induce coma. In the Drugbank database, based on studies in animal models, carisoprodol-induced muscle relaxation is associated with changes in the activity of interneurons in the spinal cord and descending reticulum located in the brain. Homatropine methylbromide (D02070) is a quaternary ammonium muscarinic acetylcholine receptor antagonist belonging to the group of medicines called anti-muscarinics. Research in the DrugBank database shows that it is used to treat duodenal or gastric ulcers or intestinal problems and prevent nausea, vomiting, and motion sickness. Meanwhile, Homatropine methylbromide is classed explicitly as Alimentary tract and metabolism in the KEGG database. These successful prediction result show that our model can provide valuable information for drug discovery and predict the potential pharmacological properties of drugs.

**TABLE 7:** Eight inferred drugs ATC class based on the DACPGTN model.

Drug ID	Chemical Name	Original ATC Class	Inferred ATC Class	Evidences
D00302	Dipyridamole	*S*_2_	${S_{3}}^{*}$	KEGG/CTD
D02070	Homatropine methylbromide	*S*_13_	${S_{1}}^{*}$	KEGG/DrugBank
D00768	Carisoprodol	*S*_9_	${S_{10}}^{*}$	DrugBank/CTD
D00652	Brinzolamide	*S*_13_	${S_{3}}^{*}$	KEGG
D00131	Disulfiram	*S*_10_, *S*_11_	${S_{1}}^{*}$ , *S*_14_	KEGG/CTD
D01192	Olopatadine hydrochloride	*S*_12_, *S*_13_	${S_{3}}^{*}$	CTD
D00314	Etidronate disodium	*S*_9_	${S_{10}}^{*}$	CTD
D00525	Pilocarpine	*S*_10_, *S*_13_	${S_{1}}^{*}$ , ${S_{4}}^{*}$ , ${S_{12}}^{*}$	CTD

^*^This symbol indicates that evidences can be found to support the chemical belonging to the ATC class.

## 5 Conclusion

Considering drug ATC code identification can play an important role in drug discovery and development, we proposed an end-to-end model DACPGTN based on graph transformer network to predict the ATC code for drugs effectively in this study. DACPGTN formulated the ATC code prediction of drugs as a multi-label classification problem. By applying transformer network, DACPGTN learned comprehensive interactions among drugs, diseases and targets to construct drug-target-disease heterogeneous networks. Moreover, DACPGTN integrated various biomedical information to obtain more representative features of drugs, diseases and targets. Based on the learned heterogeneous network and features, graph convolution network was used to obtain network embedding of drugs for drug ATC code multi-label classification task. For the drug ATC code multi-label prediction problem, we transformed it into the calculation of the difference between the score of the target class and the score of the non-target class, which solves the class-imbalance problem to a certain extent. The results of cross-validation experiments have demonstrated that DACPGTN is an effective approach to identify the ATC code of drugs, which can help the pharmacological discovery of drugs. In the future work, more high-quality data and biomedical entities can be incorporated to obtain more effective features of drugs. In addition, the performance and usefulness of the DACPGTN model can be further improved by utilizing attention-based mechanisms.

## Data Availability

The datasets for this study can be found in the https://github.com/Szhgege/DACPGTN/tree/main/data.
